# 

**Published:** 1865-07

**Authors:** 


### NOTICES.

ALBANY, N. Y.

The first regular meeting of the New York State Dental Delegation, will be held in the City of Albany, at Association Hall, on Thursday the 20th of July, commencing at 10 o'clock A. M. All members of local societies are entitled to Membership upon signing the Constitution; and any Dentist in good standing may be elected by vote of the Delegation. It is hoped that the different local societies throughout the State will send up a full representation; and that every Dentist who feels an interest in the progress of our Profession will make it a point to be present.

Your attendance is respectfully requested.

By order of the Delegation.

J. A. PERKINS, *President.* B. WOOD, *Corresponding Secretary,*

### OF THE WEST.

*Issued Monthly, at $3 a Year in Advance.*

DEVOTED TO THE INTERESTS OF THE DENTAL PROFESSION.

**EDITED BY J. TAFT**

The volume commences in January and closes in December.

The Register being issued monthly is always fresh, therefore indispensable to the practitioner who desires to keep posted up with the new inventions and improvements which are daily developed. Neither expense nor effort will be spared to give value and variety to its contents. Articles will be illustrated with well executed engravings whenever they will add to the interest or assist in explanation.

Its extensive circulation and frequency of issue makes it one of the BEST DENTAL ADVERTISING MEDIUMS in the United States.

RATES OF ADVERTISING.

One page, 1 year, $50. Half page, 1 year, $30. Quarter page, or card, 1 year $20.

All advertising bills payable in advance, or at furthest after the issue of the first number ontaining the advertisement. All transient advertisements to be paid for in advance.

☞ CONTRIBUTIONS SOLICITED.

Address, J. TAFT, or "DENTAL REGISTER," Cincinnati, Ohio.

Business Communications to

**J. TAFT, Publisher,**
No. 172 Race Street.

### ROBERTS' OS-ARTIFICIAL.

A substitute for AMALGAM in filling badly decayed teeth; and used for reacting Pivot Teeth in badly decayed roots, also for filling over SENSITIVE DENTINE to destroy sensibility, and as a non-conductor of heat, and for many other dental purposes.

For sale by all dealers in Dental Materials, and by the undersigned.

One-fourth ounce packages with directions sent by mail free of postage on receipt of $1.

C. H. ROBERTS, M. D.

**POUGHKEEPSIE, N. Y.**

